# Influences of Teacher–Child Relationships and Classroom Social Management on Child-Perceived Peer Social Experiences During Early School Years

**DOI:** 10.3389/fpsyg.2020.586991

**Published:** 2020-10-15

**Authors:** Jing Chen, Hui Jiang, Laura M. Justice, Tzu-Jung Lin, Kelly M. Purtell, Arya Ansari

**Affiliations:** ^1^Crane Center for Early Childhood Research and Policy, The Ohio State University, Columbus, OH, United States; ^2^Department of Educational Studies, The Ohio State University, Columbus, OH, United States; ^3^Department of Human Sciences, The Ohio State University, Columbus, OH, United States

**Keywords:** child-perception of peer social experiences, peer social support, peer victimization, teacher–child relationships, classroom social management

## Abstract

Interactions with teachers and peers are critical for children’s social, behavioral, and academic development in the classroom context. However, these two types of interpersonal interactions in the classroom are usually pursued via separate lines of inquiries. The current study bridges these two areas of research to examine the way in which teachers influence child-perceived peer social support and peer victimization for 2,678 children within 183 classrooms in preschool through grade three. Two levels of teacher influence are considered, namely teacher–child closeness and conflict relationships at the child-level, and teacher management of interpersonal interactions at the classroom-level. Results of multilevel regression models showed that teacher–child closeness was associated with the growth of child-perceived peer social support from fall to spring, whereas teacher–child conflict and teachers’ behavior management practices were associated with the change in child-perceived peer victimization across the academic year. These associations were unique and above and beyond the influence of children’s actual peer social interactions, including reciprocal friendships and the collective classroom reputation of peer victimization. Collectively, findings highlight the multi-faceted teacher roles in shaping children’s perceptions of their peer social experiences during the earliest years of schooling.

## Introduction

Children’s interactions with their teachers and peers are both salient features of the classroom environment and figure prominently in theories concerning children’s development and learning (Bronfenbrenner and Morris, 2006). Studies find that positive interactions with teachers and with peers and the way in which teachers manage interpersonal interactions in the classroom influence children’s concurrent and long-term social, emotional, and academic development (e.g., Kochenderfer and Ladd, 1996; Jerome et al., 2009; Hosan and Hoglund, 2017; Ladd et al., 2017). However, much of the extant literature has considered the influence of teachers and peers separately (Hughes and Im, 2016; Wang et al., 2016). Consequently, we know little about the roles of teachers in optimizing children’s perceptions of their peer social experiences and whether teacher influences are above and beyond children’s actual peer social interactions. This an important gap in knowledge because researchers have argued that children’s *perceptions* of their peer social experiences might be more predictive of their social and psychological well-being and school success than their *actual* peer interactions (Betts et al., 2013; Troop-Gordon et al., 2019; Önder et al., 2019). Thus, the current study examines multiple levels of teacher influences, including teacher–child relationships (i.e., closeness and conflict) at the child-level and teachers’ classroom management of interpersonal interactions at the classroom-level, on two aspects of peer social experiences from children’s perspective: peer social support and peer victimization.

### Significance of Child-Perceived Peer Social Experiences

Peer social support and peer victimization are two important aspects of children’s classroom experiences. Peer social support refers to supportive behaviors from peers that can enhance children’s functioning and resilience to difficulties (Bakalım and Taşdelen-Karçkay, 2016). Bakalım and Taşdelen-Karçkay argued that peer social support provides children with emotional comfort that protects children against anxiety and stress, helps them cope with difficulties via guidance and feedback. Indeed, peer social support is associated with a range of positive outcomes, including children’s motivation, attention, academic attitudes, and achievement (Coolahan et al., 2000; Bursal, 2017). Thus, peer social support is considered as a primary indicator of school adaptiveness and academic success from preschool through elementary school and above (Coolahan et al., 2000; Blandon et al., 2010).

Peer victimization, on the other hand, has been linked with school maladjustment, which refers to physical and emotional harms children receive from peers, such as being hit and teased. Studies find that peer victimization is a precursor of loneliness and school avoidance (Kochenderfer and Ladd, 1996; Buhs and Ladd, 2001) and is associated with low self-esteem, depression, external behavioral problems, and academic failure (Olweus, 1992; Alsaker, 1993; Blandon et al., 2010; Ladd et al., 2017). Researchers report that children who experience peer victimization tend to be less engaged classroom activities, which, in turn, is associated with their emotional adjustment difficulties and limits their access to opportunities and resources that are essential for social and academic development (Buhs and Ladd, 2001; Blandon et al., 2010).

Although children’s perceptions of their peer social experiences are related to their actual peer social interactions (Kochenderfer and Ladd, 1996), only a few studies have conceptually differentiated children’s perceived peer experiences from their actual peer experiences. This differentiation is important because some researchers suggest that perceptions of being supported by peers reflect children’s competency in peer interactions, which is associated with their learning behaviors and school success (Coolahan et al., 2000; Blandon et al., 2010). Specifically, in the literature of peer isolation, the distinction between objective isolation and perceived isolation has been established, with the former representing the actual quantity of peer interactions and the latter capturing loneliness or the feeling of being isolated by peers (Cacioppo and Hawkley, 2009; Danese et al., 2009).

Differentiating perceived from actual peer social experience is also meaningful because children’s perceptions might be more strongly associated with their social and emotional well-being. On the one hand, children’s perceptions of their peer social experiences can shape their self-perceptions or self-worth, which can then influence children’s social behaviors (Ogelman et al., 2019) and their levels of being liked by peers (Önder et al., 2019). Önder et al. explained that self-perception reflects one’s own competence and personality, which is established when children perceive their strengths and weaknesses when interacting with others and that children with low self-perception are likely to be passive and timid in peer interactions, which would contribute to their being less liked by peers. On the other hand, Troop-Gordon et al. (2019) discussed that support and victimization experiences in peer groups build children’s beliefs about peers, which, according to social information processing theories, would shape their behavioral and emotional responses to future interpersonal events. Some suggest that perceived isolation tends to result in more severe and enduring consequences than objective isolation, because the perceptions of being isolated can alter individuals’ social reasoning and information processing (Cacioppo and Hawkley, 2009; Danese et al., 2009). Specifically, Cacioppo and Hawkley explained that the perception of being isolated by peers may trigger children’s confirmatory and memorial bias and can lead to their negative interpretations of peers’ social moves, which in turn may contribute to children’s misbehaviors and emotional maladaptiveness. Hence, although perceived and actual peer social experiences are rarely distinguished in the broader sense of peer social experience, it stands to reason that perceived peer social support and perceived peer victimization would shape children’s understandings about themselves and about others. Therefore, there is a need to examine factors that may influence children’s perceptions of their peer social experiences.

### Teacher Influences on Peer Social Experiences

Besides peers, teachers represent another key dimension of classroom ecology (Hamre and Pianta, 2001; Jerome et al., 2009). As noted earlier, however, interactions with teachers and interactions with peers tend to be discussed separately (Hughes and Im, 2016; Wang et al., 2016), except for only a few studies as elaborated below; such work has suggested that teachers’ relationships with individual children and their classroom social management can shape children’s peer social experiences in the classroom.

For individual children, their interactions with teachers matter to their social experiences with peers. This is because teacher–child interactions can be observed by all classmates, which helps classmates draw inferences about children’s attributes and likeability and form a classroom consensus about children’s reputations (Hughes and Im, 2016). Further, teacher–child closeness is grounded in positive interactions, such as warm and open communications, between a teacher and a child (Birch and Ladd, 1997), which forms a secure base for children to feel being cared and connected to the classroom environment. Teacher–child closeness is associated with children’s engagement in classroom activities and their social competences and peer acceptance (e.g., Birch and Ladd, 1997; Pianta and Stuhlman, 2004; Hall-Lande et al., 2007; Gest and Rodkin, 2011). Children with close relationships with teachers may also receive greater support from teachers, which contributes to their social and academic development (Hamre and Pianta, 2001). On the contrary, teacher–child conflicts contribute to peer disliking as well as school avoidance, externalizing behaviors, and decreased prosocial behaviors and cooperation (Hamre and Pianta, 2001; Hughes and Im, 2016).

At the classroom-level, teachers’ classroom management of interpersonal interactions (i.e., classroom social management) serves to shape children’s peer social experience. Classroom social management is a challenge and critical task for teachers, which requires them to be aware of children’s social needs and to afford developmental opportunities for children to positively interact with peers from diverse backgrounds (Farmer et al., 2019). A commonly used tool to capture classroom social management is the *Classroom Assessment Scoring System* (CLASS; Hamre and Pianta, 2007; Pianta et al., 2008; Downer et al., 2012), which features three domains of classroom management based on social and instructional interpersonal interactions (i.e., emotional support, classroom organization, and instructional support). These three domains are further categorized into nine dimensions. The current study includes four dimensions that mainly focus on the social aspect of interactional interactions, naming *positive climate*, which refers to interactions between teachers and children and among children that feature enthusiasm, enjoyment, and respect; *negative climate*, which refers to classroom interpersonal interactions that involve anger, aggression, or harshness; *teacher sensitivity*, which represent the extent to which teachers provide comfort, reassurance, and encouragement based on individual children’s needs; and *behavior management*, which refers to teachers’ effectiveness in preventing and redirecting children’s misbehaviors. Warm and sensitive interactions with teachers and well-managed classrooms promote classroom inclusiveness and facilitate social connections among children, through which children develop social and emotional competences, reduce problematic behaviors, and become less vulnerable to peer victimizations (Hamre and Pianta, 2001; Cappella and Neal, 2012; Downer et al., 2012).

Although teachers can influence children’s peer social experiences via multiple avenues as reviewed above, few studies have taken into account different levels of teacher influences simultaneously. Farmer et al. (2019) discussed that teachers are not only members in the classroom society interacting directly with individual children, but, at the same time, they also are leaders who act as an authority and a facilitator to manage classroom dynamics and to ensure children following the rules. Hence, the current study aims to capture teachers’ multi-faceted roles to have a more comprehensive understanding of teacher influence on children’s peer social experiences in the classroom.

### The Current Study

The current study focuses on children from preschool through grade three; during these grades, positive peer experiences provide essential support to children’s development and learning, whereas peer victimization occurs relatively more often than that in the later grades (Kochenderfer and Ladd, 1996; Ladd et al., 2017). Thus, there is a need to investigate teacher roles in managing classroom social dynamics during children’s primary years of schooling.

Although there has been some research examining certain teacher influence on children’s peer social experiences, it is not clear whether teacher influences operate above and beyond the influence of children’s actual peer social interactions. For the purpose of this study, children’s actual peer interactions were operationalized as the number of reciprocal friendships and their classroom reputation of peer victimization. Friendship is considered as the most important source of peer support, which provides children with a context for skill acquisition and development and helps children to validate their shared beliefs and identifies (Ladd et al., 1996; Gifford-Smith and Brownell, 2003). Further, compared to unilateral friendships (i.e., one child identifies the other as a friend but not vice versa), reciprocal friendships (i.e., children mutually identify each other as friends) tend to have higher quality, are more stable, and, therefore provide greater peer support (e.g., Quinn and Hennessy, 2010). Classroom reputation of peer victimization reflects the consensus among all classmates about the extent of harassment one experiences from peers. Hughes and Im (2016) discussed that children’s disliking of a child tends to go beyond dyadic antipathy and would be contributed greater by group-based reputation based on shared observations. Both reciprocal friendship and classroom reputation of peer victimization triangulate the perceptions from both children and peers, which, therefore, would be less biased by individuals’ opinions.

In all, the current study aims to examine multiple levels of teacher influence on child-perceived peer social support and peer victimization in the spring of the academic year when controlling for those in the fall. Teacher influences include teachers’ closeness and conflict with individual children and their classroom social management at the classroom-level as represented by observations of positive climate, negative climate, teacher sensitivity, and behavior management. A sub-aim is to determine whether the above teacher influences on children-perceived social experiences are unique and operate beyond the influence of their actual peer interactions manifested as the number of reciprocal friendships and classroom reputation of peer victimization.

## Materials and Methods

### Participants

This study is part of a large federally funded project focused on advancing understanding of early childhood learning experiences from preschool (pre-kindergarten) to third grade. The study sample consisted of two cohorts of participants, recruited from two large school districts in a Midwestern state. Recruitment procedures were carried out in accordance with protocols to protect human subjects as approved by the Institutional Review Board (IRB) of the university.

Before the school year started, informational sessions were held in schools located within district borders to recruit teachers. All children in classrooms taught by participating teachers were eligible to enroll, and consent packets were sent home via backpack mail. Most participants were recruited in the fall, although additional preschool classrooms were added in winter and spring to meet recruitment goals. Consented teachers were asked to complete questionnaires about their classrooms, their children, their teaching practices, and their own background information. Consented children were administered direct assessments in fall and spring of the school year.

The sample included 43 schools, 183 classrooms, and 2,678 consented children. As summarized in Table 1, 50% of the participating children were girls, 66% were White, and 13% were Hispanic/Latino(a). Twelve percent of the children came from households that primarily spoke a language other than English and 10% of children had identified disabilities. Annual family income was distributed bimodally with 27% of the participating families falling in the lowest income bracket ($30,000 or lower) and 31% in the highest income bracket ($120,001 or higher). Forty-five percent of the children’s mothers completed 4-year college education or higher. At the classroom level, an average classroom had 22 children (range = 12–29). Teachers were mostly female (97%), White (96%), and non-Hispanic (99%). On average, they were 38 years old with 13 years of teaching experience. Ninety-four percent of the teachers had a bachelor’s degree or higher, and 82% had a teaching certificate.

**TABLE 1:** Sample description.

Variable	Valid *N*	% missing	Mean, %	*SD*	Range
**Child and family characteristics**					
School district	2678	0.0			
District 1			62.1%		
District 2			37.9%		
Grade level	2678	0.0			
Preschool			21.7%		
Kindergarten			24.7%		
First grade			17.8%		
Second grade			18.9%		
Third grade			16.9%		
Child gender	2659	0.7			
Female			49.5%		
Male			50.5%		
Child race	2628	1.9			
White/Caucasian (non-multiracial)			66.1%		
Black/African American (non-multiracial)			8.0%		
Asian (non-multiracial)			6.3%		
Other (non-multiracial)			7.9%		
Multiracial			11.8%		
Child is Hispanic	2637	1.5	12.8%		
Child has an IEP in spring	2450	8.5	10.1%		
Primary language spoken at home is English	2649	1.1	87.9%		
Annual household income	2564	4.3			
<$30,001			27.2%		
$30,001–$60,000			16.8%		
$60,001–$90,000			12.9%		
$90,001–$120,000			12.0%		
>$120,000			31.1%		
Mother’s highest level of education	2619	2.2			
Less than high school diploma			10.3%		
High school diploma or GED			31.8%		
Associate degree			12.8%		
Bachelor’s degree			24.8%		
Graduate or professional degree			20.3%		
Child age in fall (in months)	2650	1.0	78.16	18.37	25–124
Number of people in household	2026	24.3	4.51	1.23	2–9+
Number of children (age < 18) in household	2026	24.3	2.47	1.12	1–9+
**Classroom and teacher characteristics**					
School district	183	0.0			
District 1			64.5%		
District 2			35.5%		
Grade level	183	0.0			
Preschool			25.7%		
Kindergarten			25.7%		
First grade			15.8%		
Second grade			16.9%		
Third grade			15.8%		
Teacher gender	178	2.7			
Female			97.2%		
Male			2.8%		
Teacher race	175	4.4			
White/Caucasian (non-multiracial)			96.0%		
Black/African American (non-multiracial)			2.3%		
Other (non-multiracial) and multiracial			1.7%		
Teacher ethnicity [1 = Hispanic/Latino(a)]	174	4.9	1.1%		
Certification status (1 = Yes)	169	7.7	82.8%		
Teacher’s highest level of education	174	4.9			
High school diploma or GED			1.1%		
Some college credit, no degree			2.3%		
Associate degree			2.3%		
Bachelor’s degree			35.1%		
Master’s degree			59.2%		
Teacher age (in years)	179	2.2	37.66	9.05	22–60
Teaching experience (in years)	173	5.5	13.39	8.15	2–36
Number of children in classroom	178	2.7	21.90	3.99	12–29

*Means are reported for continuous variables and percentages reported for categorical variables.*

### Measures

To address the aims of the current study, we included measures of child-perceived peer social experiences, teacher–child relationships, classroom social management, and actual peer social interactions. Children’s family background and demographic information were collected from caregiver and teacher questionnaires at the beginning of the school year.

#### Child-Perceived Peer Social Experiences

In fall and spring of the school year, one-on-one child interviews were conducted by trained research staff in quiet areas of the school hallway, and responses were recorded using a tablet in accordance with the approved study protocols. Based on previous studies of peer relationship and children’s school adjustment (Asher et al., 1984; Ladd, 1990; Kochenderfer and Ladd, 1996; Waters et al., 2012), the research team developed measures of perceived peer social support comprising a total of 11 items (e.g., “How often would kids in your class help you if you are hurt?” and “How often would kids in your class tell you you’re good at things?”) and perceived peer victimization consisting of four items (e.g., “Does anyone in your class ever hit you?” and “Does anyone in your class ever say mean things to you?”). All items used a three-point frequency scale (0 = *Never*, 1 = *Sometimes*, 2 = *A lot*), and the internal consistency (Cronbach’s alpha) ranged from 0.75 to 0.78 across scales and time points. The responses from items on the same scale were averaged to create composite scores for each child. In the analysis, spring scores were used as outcomes, and fall scores were included as covariates.

#### Teacher–Child Relationships

In the fall, teachers reported on their closeness and conflict with each child using the *Student–Teacher Relationship Scale* (Pianta, 2001). The closeness subscale included seven items (e.g., “I share an affectionate, warm relationship with this child” and “If upset, this child will seek comfort from me”) and the conflict subscale contained eight items (e.g., “This child and I always seem to be struggling with each other” and “Dealing with this child drains my energy”). All items used a five-point Likert-type scale (0 = *Definitely does not apply*, 4 = *Definitely applies*) and the scales demonstrated strong internal consistency (alphas ranged from 0.88 to 0.94). For analysis, the mean score of each subscale of the teacher–child relationship was calculated for each child.

#### Classroom Social Management

Teacher’s classroom social management was captured in the winter with the *Classroom Assessment Scoring System* (CLASS, Pianta et al., 2008). As noted earlier, although the original CLASS includes nine dimensions, the current study focuses on four dimensions mainly from the social domain, including (1) *positive climate*, which reflects the warmth, respect, and enjoyment communicated by verbal and non-verbal interactions, (2) *negative climate*, which assesses the overall level of expressed negativity among teachers and children in the classroom, (3) *teacher sensitivity*, which refers to the teacher’s awareness and responsiveness to the various needs of individual children and the entire class, and (4) *behavior management*, which encompasses the teacher’s use of clear behavioral expectations and effective methods to prevent and redirect misbehavior. In each classroom, trained and reliable research staff conducted two 30-min observation cycles, where observers live-coded the teacher’s practice or behavior as it contributed to the overall classroom environment on scales of 1 to 7 (1 = *minimally characteristic*, 7 = *highly characteristic*). Composite scores for each dimension were created by averaging across the two cycles. To ensure reliability, research staff completed extensive training sessions before entering the field, and ongoing quality checks were conducted via biweekly drift meetings. In addition, 20% of all in-field observations were double-coded, and inter-rater agreement (i.e., two coders scored within one point of difference on the same dimension) ranged from 0.90 to 0.92.

#### Peer Social Interactions

Peer social interactions including *reciprocal friendships* and *classroom reputation of peer victimization* were collected in the spring based on a peer nomination approach (Parkhurst and Asher, 1992), which has been found valid for children as young as preschoolers (Daniel et al., 2016; Chen et al., 2020). We asked children to identify classmates “*who are your best friends*” and “*who gets picked on or teased?*” Preschoolers were presented with a photo roster of all children in their classrooms to facilitate the nomination, while older children were provided a list of names of their classmates. For each child, we counted the number of reciprocal friendships when the child and classmates mutually nominated each other as best friends; classroom reputation of peer victimization was represented by the frequency at which the child was nominated by classmates as someone who gets picked on or teased. Children’s raw scores were standardized by dividing classroom size minus one, the maximum possible value, to allow the indices to be compared across classrooms.

### Analytical Approach

We employed multilevel regression models to investigate the effects of teacher influence on children’s perception of peer social experiences, given that children (level-1) were nested within classrooms (level-2). Two outcomes were examined, namely the child-perceived peer social support and child-perceived peer victimization in the spring. For each outcome, we first ran unconditional multilevel models where child outcomes were clustered by classrooms, to determine the percentage of observed variance attributable to classroom differences. Second, we fitted conditional multilevel models (Model 1), examining the association between teacher–child relationships and teacher classroom management and child-perceived peer social experiences, controlling for the pretest scores (i.e., child-perceived peer social experiences in the fall). Other controlled variables included child gender, disability status reported by teachers in spring, child race reported by caregivers (dichotomized into White vs. non-White), grade level, and school district. Finally, we included actual peer social interactions (i.e., reciprocal friendship and classroom reputation of peer victimization) as covariates to test whether teacher influences contribute to children’s perceptions above and beyond their actual peer social interactions (Model 2). All models were fit in R with the *lmer* package (Bates et al., 2015) with maximum likelihood estimation. Missing data were list-wise deleted. The proportion of missing for each variable is reported in Table 1.

## Results

As shown in Table 2, children generally perceived that they had some peer social support, both in fall and in spring (mean = 1.32 and 1.35) with 75–79% reporting scores between 1 (*Sometimes*) and 2 (*A lot*). The mean of child-perceived victimization was 0.44 and 0.53 in the fall and spring, respectively, with 32–34% of children reporting never experiencing peer victimization. A little over one-half of children (55% in fall and 51% in spring), however, perceived experiencing some victimization, with scores greater than 0 (*Never*) but less than 1 (*Sometimes*). In terms of teacher–child relationships, teachers reported moderate to high levels of closeness (*M* = 3.13 out of 4) and low levels of conflict (*M* = 0.63 out of 4). Additionally, the classrooms were rated as having moderate quality in terms of teacher sensitivity (*M* = 4.65 out of 7), behavior management (*M* = 5.42 out of 7), and positive climate (*M* = 5.52 out of 7), and were scored very high in the area of negative climate (suggesting the absence of negativity; *M* = 6.92 out of 7). Finally, in terms of actual peer social interactions, children had reciprocal friendships with 8% of their classmates (range = 0–38%) and were nominated as “being picked on or teased” by 4% of their classmates (range = 0–80%).

**TABLE 2:** Descriptives of key study variables.

Variable	*N*	% missing	Mean	*SD*	Range
**Child-perceived peer social experiences**					
Child-perceived peer support fall	2214	17.3	1.32	0.42	0.00–2.00
Child-perceived peer support spring	2443	8.8	1.35	0.39	0.00–2.00
Child-perceived peer victimization fall	2234	16.6	0.44	0.51	0.00–2.00
Child-perceived peer victimization spring	2457	8.3	0.53	0.54	0.00–2.00
**Actual peer social interactions (standardized)**					
Reciprocal friends	2461	8.1	0.08	0.07	0.00–0.38
Reputation of peer victimization	2662	0.6	0.04	0.06	0.00–0.80
**Teacher–child relationships**					
Teacher–child closeness	2293	14.4	3.13	0.69	0.00–4.00
Teacher–child conflict	2293	14.4	0.63	0.80	0.00–4.00
**Classroom social management**					
CLASS behavior management	179	2.2	5.42	0.78	3.00–7.00
CLASS teacher sensitivity	179	2.2	4.65	1.02	2.00–7.00
CLASS positive climate	179	2.2	5.52	0.84	2.50–7.00
CLASS negative climate	179	2.2	6.92	0.24	5.50–7.00

Pairwise correlations are presented in Table 3. There was a moderate correlation between child-perceived peer experiences in the fall and the spring (0.41–0.55). Child-perceived peer victimization was negatively correlated with teachers’ behavior management scores (−0.25 to −0.20), and child-perceived peer victimization in the spring was also negatively correlated with teachers’ ability to promote a positive climate (−0.16). In addition, teacher–child closeness and conflict were negatively correlated (−0.27), and the four CLASS indices were positively correlated (0.17–0.70).

**TABLE 3:** Pearson correlation coefficients among key study variables.

	1	2	3	4	5	6	7	8	9	10	11	12
**Child-perceived peer social experiences**												
1. Peer support fall	−											
2. Peer support spring	0.55^∗^	−										
3. Peer victimization fall	0.18	0.06	−									
4. Peer victimization spring	–0.13	0.20^∗^	0.41^∗^	−								
**Actual peer social interactions**												
5. Reciprocal friends	0.07	0.10	–0.11	–0.12	−							
6. Reputation of peer victimization	–0.07	0.03	–0.02	0.11	–0.13	−						
**Teacher–child relationship**												
7. Teacher–child closeness	0.16	0.13	0.04	–0.09	0.05	–0.13	−					
8. Teacher–child conflict	–0.18	–0.12	0.14	0.16	–0.01	0.15	−0.27^∗^	−				
**Classroom social management**												
9. CLASS behavior management	–0.07	–0.14	−0.25^∗^	−0.20^∗^	0.07	–0.12	–0.07	0.12	−			
10. CLASS teacher sensitivity	–0.07	0.01	–0.13	–0.06	–0.05	–0.04	–0.06	0.07	0.38^∗^	−		
11. CLASS positive climate	–0.07	–0.06	–0.12	−0.16^∗^	0.08	–0.08	–0.10	–0.07	0.70^∗^	0.51^∗^	–	
12. CLASS negative climate	0.03	0.07	–0.15	–0.11	0.06	–0.08	–0.02	0.05	0.42^∗^	0.17^∗^	0.40^∗^	–

*p < 0.05.

### Teacher Influences on Child-Perceived Peer Social Experiences

The primary aim of the current study was to examine the associations between teacher–child relationships and teachers’ classroom social management and two aspects of child-perceived peer social experiences in the spring: peer social support and peer victimization. The unconditional model (Model 0, output not presented) showed that for perceived peer social support, 3% of the variance (<0.01) was attributable to differences between classrooms, and 97% (0.15) was due to individual differences. For perceived peer victimization, 14% of the variance (0.04) was accountable by classroom-level differences, while 86% of the variation (0.25) was between children.

Next, our focal teacher predictors of interest were included in Model 1 (Table 4). Results showed that, after controlling for fall responses on child-perceived peer social experiences and other covariates, teacher–child closeness significantly predicted child-perceived peer social support (*b* = 0.04, *p* < 0.01) and teacher–child conflict predicted child-perceived peer victimization (*b* = 0.10, *p* < 0.001). Specifically, with one additional unit increase in teacher–child closeness (on a scale of 0 to 4), child-perceived peer social support was expected to increase by 0.04 units (on a scale of 0 to 2). With one unit increase in teacher–child conflict, child-perceived peer victimization was expected to increase by 0.10 units. At the classroom level, teachers’ behavior management was negatively associated with child-perceived peer victimization (*b* = −0.07, *p* < 0.05). A unit increase in behavior management (on a scale of 1 to 7) was associated with 0.07 unit of *decrease* in child-perceived peer victimization. Collectively, Model 1 accounted for approximately 20% of the variance for both of the outcome variables at the child level, and over 70% of the variance at the classroom level for child-perceived peer victimization. Almost no extra classroom-level variance for child-perceived peer social support was accounted for by the above variables, which might be because there was originally little classroom-level variance (4%) in total as suggested by the unconditional model.

**TABLE 4:** Predicting child-perceived peer social support and peer victimization in spring: Model 1.

	Peer social support		Peer victimization	
		
	Coefficient	SE	Coefficient	SE
**Pretest**				
Child-perceived peer social support fall	0.34***	0.02		
Child-perceived peer victimization fall			0.37***	0.02
**Demographics**				
Preschool vs. K	–0.00	0.03	–0.04	0.05
Grade 1/2/3 vs. K	0.03	0.03	–0.03	0.04
District 1 vs. 2	–0.01	0.02	0.07*	0.03
Child is a girl	0.01	0.02	0.04	0.02
Child has a disability (spring)	−0.07*	0.03	0.02	0.03
Child is white	0.03	0.02	0.00	0.03
**Teacher–child relationship and teacher practice**				
Teacher–child closeness (cmc)	0.04**	0.01	0.04	0.02
Teacher–child conflict (cmc)	–0.02	0.01	0.10***	0.02
CLASS behavior management	0.00	0.02	−0.07*	0.03
CLASS teacher sensitivity	–0.00	0.01	0.01	0.02
CLASS positive climate	0.02	0.02	–0.00	0.03
CLASS negative climate	0.02	0.05	–0.07	0.07
**Model information**				
AIC	1493.19		2564.03	
BIC	1582.41		2653.41	
Log likelihood	–730.60		–1266.02	
Number of children	1951		1971	
Number of classrooms	163		163	
Level-2 variance (intercept)	0.00		0.01	
Level-1 variance (residual)	0.12		0.20	

cmc, class-mean centered. ***p < 0.001, **p < 0.01, *p < 0.05.

Finally, to determine whether the associations reported above were unique, we included children’s actual peer social interactions in Model 2 (Table 5), which were operationalized as reciprocal friendships and classroom reputation of peer victimization. Results showed that even though reciprocal friendship was a strong predictor of child-perceived peer social support (*b* = 0.74, *p* < 0.001) and classroom reputation of peer victimization was predictive of self-perceived peer victimization (*b* = 0.94, *p* < 0.001), the above-reported association associations were stable and remained significant.

**TABLE 5:** Predicting child-perceived peer social support and peer victimization in spring: Model 2.

	Peer social support		Peer victimization	
		
	Coefficient	SE	Coefficient	SE
**Pretest**				
Child-perceived peer social support fall	0.33***	0.02		
Child-perceived peer victimization fall			0.37***	0.02
**Demographics**				
Preschool vs. K	–0.01	0.03	–0.06	0.05
Grade 1/2/3 vs. K	0.01	0.02	–0.03	0.04
District 1 vs. 2	0.00	0.02	0.06*	0.03
Child is a girl	0.02	0.02	0.04	0.02
Child has a disability (spring)	−0.06*	0.03	0.01	0.03
Child is white	0.02	0.02	0.00	0.03
**Teacher–child relationship and teacher practice**				
Teacher–child closeness (cmc)	0.04**	0.01	0.04	0.02
Teacher–child conflict (cmc)	–0.01	0.01	0.09***	0.02
CLASS behavior management	–0.00	0.02	−0.07*	0.03
CLASS teacher sensitivity	0.00	0.01	0.01	0.02
CLASS positive climate	0.02	0.02	0.01	0.03
CLASS negative climate	0.01	0.05	–0.07	0.07
Peer social interactions				
Reciprocal friends	0.74***	0.11	–0.09	0.15
Reputation of peer victimization	–0.17	0.12	0.94***	0.16
**Model information**				
AIC	1436.71		2508.44	
BIC	1536.86		2608.76	
Log likelihood	–700.36		–1236.22	
Number of children	1927		1946	
Number of classrooms	163		163	
Level-2 variance (intercept)	0.00		0.01	
Level-1 variance (residual)	0.11		0.19	

cmc, class-mean centered. ***p < 0.001, **p < 0.01, *p < 0.05.

## Discussion

The current study examined the interplay among teachers, children, and peers as actors in the classroom social ecology during early school years. Specifically, we focused on the influences of teacher–child closeness and conflict and teacher’s classroom social management on child-perceived peer social support and peer victimization. The current study expands on the existing literature by, first, simultaneously taking into account teachers’ roles as classroom members who form closeness and conflict with individual children and as leaders who shape classroom social dynamics, and, second, by further highlighting the critical roles of teachers in shaping children’s perceptions of their peer social experiences, after controlling for children’s actual peer social interactions. The major findings are discussed below.

First, it is evidenced that teacher influence at the individual-level and that at the classroom-level are unique, and that each contributes to child-perceived peer social experiences. In terms of the relationships between teacher and individual children, our findings showed that teacher-reported closeness and conflict with children in the fall contributed to peer social support and peer victimization perceived by children in the spring respectively, controlling for the fall scores. This finding indicates that children with close relationships with teachers tend to feel more socially supported by peers and that children who have conflicts with teachers tend to experience increased perceived peer victimization over the academic year. These findings are in line with the literature that teacher–child interactions broadcast children’s attributes and likability to classmates who observe the interactions (Hughes and Im, 2016), which foster a classroom consensus regarding children’s reputations and therefore influence classmates’ interactions with the children. It is also likely that positive teacher–child relationships can promote children’s cooperative engagement in classroom activities and improves their social competence, while with negative teacher–child relationships, children may avoid school and demonstrate more externalizing behavior problems and less prosocial behaviors during interpersonal interactions (Hamre and Pianta, 2001; Hughes and Im, 2016).

Second, regarding teachers’ classroom social management, our findings showed that better behavior management in the fall was associated with less peer victimization as perceived by children in the spring controlling for the fall scores. This finding suggests that in classrooms where misbehaviors are better managed and redirected, child-perceived peer victimization decreases over time. This finding is aligned with literature showing that well-managed classrooms are associated with greater social and academic development and with reduction of behavior problems (Emmer and Stough, 2001; Downer et al., 2012). Kochenderfer-Ladd and Pelletier (2008) further discussed that, when teachers do not consider bullying as a normative behavior in the classroom, they would be more likely to intervene toward negative peer social interactions rather than expecting the victims to handle the incidences on their own, which has been found associated with lower levels of peer victimization in the classroom.

However, it is surprising that the other classroom social management indicators (i.e., teacher sensitivity, positive climate, and negative climate) were not found to be positively associated with child-perceived peer social experiences in the current study. It might be that the influence of teacher sensitivity and classroom climate on children’s classroom social experiences might be more indirect than behavioral management and could take a longer time to alter children’s peer social experiences. Another possibility from the measurement perspective is that, as reported in the result section, there was minimal variance at the classroom-level in the unconditional model when predicting children-perceived peer social support, which left little room for the classroom-level teacher influences to show predictive effect. Future research may apply a more refined tool to assess these aspects of the classroom ecology.

A third major finding is that teacher influences on children’s perceptions of their social experiences operate in a manner that is unique and beyond children’s actual peer social interactions. Specifically, for children who are similar in the number of reciprocal friendships and in the collective classroom reputation of peer victimization, those who have close relationships with their teachers perceived having greater peer social support, whereas those who had conflicted relationships with their teachers perceived greater peer victimization. Also, those in classrooms with better behavior management perceived less peer victimization.

Children’s perceptions of their peer social experiences emerge based on their social interactions, which then may reflect their self-evaluation of social competence as well as beliefs about peers (Coolahan et al., 2000; Blandon et al., 2010). Our results indicate that as a member and an authoritative figure in the classroom, teachers play a critical role in shaping children’s beliefs about their own strengths and weakness in social interactions and about the classroom social environment, which operates uniquely beyond the influence of children’s actual peer social interactions. It is possible that, independent from actual interactions with peers, positive relationships with teachers and well-managed classrooms can enhance children’s sense of connectiveness with classmates, which improves their social competence in engaging in peer social interactions (Hughes and Im, 2016), and can promote the classroom inclusiveness; in turn, this may reduce problematic social behaviors and help children become less vulnerable to peer victimizations (Cappella and Neal, 2012). However, the current study does not draw causal inferences. Future study is needed to examine the mechanism and dynamic relations among teachers, peer social interactions, and children’s perceptions of their peer social experiences.

Despite these contributions to the literature, there are a few limitations in the current study. First, teacher–child relationships were assessed at a single time point. However, these relationships may vary across the academic year, as suggested by Hughes and Im (2016) who showed that the average 1-year stability of teacher–child closeness and conflict were 0.38 and 0.57 in elementary classrooms. Similarly, although children’s perceptions of peer social experiences were assessed in the fall and spring and classroom social management was observed multiple times in the winter, it is necessary for future studies to account for the change throughout an academic year in terms of children’s perceived classroom social experiences and teachers’ classroom social management. Second, at the classroom-level, teachers can shape classroom interpersonal interactions through many other ways besides classroom social management, such as seating arrangements, grouping strategies, types of activities, and responsibilities afforded to children (Farmer et al., 2019). While the current study has taken into account multiple levels of teacher influences, future research may take a more systematic and comprehensive view when examining teacher influences on classroom social dynamics. Third, when representing children’s actual peer social interactions, although the current study tried to select the most representative indicators (e.g., reciprocal friendships and classroom reputation of peer victimization), other aspects of peer social interactions can contribute to perceived peer social support, such as peer acceptance, peer rejection, and peer isolation. Future research may consider applying a latent-variable approach to account for different aspects of peer social interactions when representing children’s actual social experiences. Fourth, children’s perceptions provide a unique perspective of their peer social experiences. However, their perceptions can be biased, and so can teacher reports of their relationships with children. Future studies may consider using more objective measures to capture peer social experience and teacher–child relationships. Finally, caution is warranted when generalizing findings from the current study. Although the study sample represented a wide range of families from diverse backgrounds, families were drawn from two school districts in a single Midwestern state in the United States. Additionally, teachers who were willing to participate in this study and to be observed by researchers might have demonstrated relatively higher classroom social management skills considering the majority of them had a bachelor’s degree or higher. Accordingly, replication with different samples, measures, and methods is an important future direction.

In all, the current study demonstrated that teachers can influence children’s perceptions of their peer social experiences simultaneously through their closeness and conflict with individual children and through their classroom social management. Additionally, such teacher influences on children’s perceptions are unique from children’s actual peer social interactions. Findings underscore the need for teachers to develop close relationships with individual children and to eliminate conflict with them. As Hughes and Im (2016) suggested, although it is understandable that teachers might report conflict with children who have problem behaviors, teachers are encouraged to provide support to these children so as to optimize their classroom experiences. Beyond interactions with individual children, as the leaders in the classrooms, managing and redirecting misbehaviors can improve the quality of interpersonal interactions and reduce negative peer social experiences perceived by children. In sum, the current study highlights the multi-faceted roles of teachers in shaping children’s classroom experiences and the classroom social ecology during the earliest years of schooling.

## Data Availability Statement

The datasets generated for this study are available on request to the corresponding author.

## Ethics Statement

The studies involving human participants were reviewed and approved by The Ohio State University. Written informed consent to participate in this study was provided by the participants’ legal guardian/next of kin.

## Author Contributions

JC conceptualized this study, conducted the analysis, and drafted the manuscript. HJ wrote the method and results sections. LJ, T-JL, KP, and AA provided critical review of the manuscript. LJ, T-JL, and KP acquired the financial support for the project leading to this publication. All authors read and approved the submitted version of the manuscript.

## Conflict of Interest

The authors declare that the research was conducted in the absence of any commercial or financial relationships that could be construed as a potential conflict of interest.
